# Integrated Proteomic and Phosphoproteomics Analysis of DKK3 Signaling Reveals Activated Kinase in the Most Aggressive Gallbladder Cancer

**DOI:** 10.3390/cells10030511

**Published:** 2021-02-28

**Authors:** Kirti Gondkar, Gajanan Sathe, Neha Joshi, Bipin Nair, Akhilesh Pandey, Prashant Kumar

**Affiliations:** 1 Institute of Bioinformatics, International Tech Park, Bangalore 560066, India; kirti@ibioinformatics.org (K.G.); gajanan@ibioinformatics.org (G.S.); neha@ibioinformatics.org (N.J.); 2 Amrita School of Biotechnology, Amrita Vishwa Vidyapeetham, Kollam 690525, India; bipin@am.amrita.edu; 3 Center for Molecular Medicine, National Institute of Mental Health and Neurosciences (NIMHANS), Hosur Road, Bangalore 560029, India; pandey.akhilesh@mayo.edu; 4 Manipal Academy of Higher Education (MAHE), Manipal 576104, India; 5 Department of Laboratory Medicine and Pathology, Mayo Clinic, Rochester, MN 55905, USA; 6 Center for Individualized Medicine, Mayo Clinic, Rochester, MN 55905, USA

**Keywords:** dickkopf homologue 3, REIC, GBC, serine-threonine phospho-proteomics

## Abstract

DKK3 is a secreted protein, which belongs to a family of Wnt antagonists and acts as a potential tumor suppressor in gallbladder cancer. To further understand its tumor suppressor functions, we overexpressed DKK3 in 3 GBC cell lines. We have employed high-resolution mass spectrometry and tandem mass tag (TMT) multiplexing technology along with immobilized metal affinity chromatography to enrich phosphopeptides to check the downstream regulators. In this study, we reported for the first time the alteration in the phosphorylation of 14 kinases upon DKK3 overexpression. In addition, we observed DKK3 induced hyper phosphorylation of 2 phosphatases: PPP1R12A and PTPRA, which have not been reported previously. Canonical pathway analysis of altered molecules indicated differential enrichment of signaling cascades upon DKK3 overexpression in all the 3 cell lines. Protein kinase A signaling, Sirtuin signaling pathway, and Cell Cycle Control of Chromosomal Replication were observed to be differentially activated in the GBC cell lines. Our study revealed, DKK3 overexpression has differential effect based on the aggressive behavior of the cell lines. This study expands the understanding of DKK3-mediated signaling events and can be used as a primary factor for understanding the complex nature of this molecule.

## 1. Introduction

Dickoppf homologue 3 (DKK3), also known as reduced expression in immortalized cells (REIC) belongs to a family of secreted glycoproteins dickoppfs. The family of dickoppfs are majorly involved in embryonic development through its interaction and regulation of the Wnt signaling pathway [1]. Multiple studies, based on distinct criteria, have identified DKK3 as a divergent member of dickkopf family [1,2,3]. Unlike other family members, DKK3 is less known to be involved in Wnt signaling regulation [4] and the association of DKK3 with Wnt signaling has been poorly understood. It has been identified that DKK3 downregulates key proteins in non-canonical Wnt pathway such as c-Jun N-terminal kinase (JNK), Ca^2+^/calmodulin-dependent protein kinase II (CAMKII), and histone deacetylase 4 (HDAC4) in familial dilated cardiomyopathy [5]. In HEK293 and Muller glia MIO-M1 cell lines it is shown that DKK3 activates Wnt signaling by interacting with Krm1/2 [4]. In past years, DKK3 has been identified in modulating numerous cellular functions like immune regulatory pathways, malignancies, neurogenesis, etc. Thus, raising the exciting possibility of DKK3 involvement in multiple critical cellular processes. DKK3 overexpression has led to induction of apoptosis, and inhibition of cell migration and invasion in SQ-5 and KJ cells [6]. DKK3 overexpression reduced tumor angiogenesis in ovarian cancer by interfering with VEGFR-2/Akt/mTOR phosphorylation mediated by B2 microglobulin. Several studies also focused on the overexpression effects of DKK3 using cell-based assays [7,8,9]. Although these studies are adding knowledge on DKK3 signaling and its role in cellular level, there is unmet need to study DKK3 signaling in the systematic manner on global scale.

Proteins account for the larger part of the cell molecular machinery and as a part of organized functional modules and networks. It performs various cellular activities which determine the cell fate [10]. In recent years, mass spectrometry-based proteomics has been widely employed for the understanding the molecular and cellular signaling [11]. The alterations in protein expression could play the important role in the disease development and progression [12]. Using quantitative proteomics approaches, these variations at protein level can be detected and measured which provides valuable information about the understanding of the molecular mechanisms involved. Post-translational modifications (PTM) are key regulators of the protein function. It plays a pivotal role in understanding the cellular mechanisms [13]. Protein phosphorylation is one of the important and most commonly studied post- translational modification PTM [14]. Activation and deactivation of many enzymes and proteins by kinases and phosphatases play important role in governing signaling, adhesion, and cell communication mechanisms [15,16,17].

Gallbladder cancer (GBC) is highly invasive and molecularly heterogenous cancer. The molecular mechanisms underlying the progression of this disease are not well studied. Isocitrate dehydrogenase (IDH), fibroblast growth factor receptor (FGFR), epidermal growth factor receptor (ERBB), KRAS, ROS1 remain to be the most promising therapeutic targets to date [18,19]. Identification of molecular factors implied in prognosis of this disease remains an unmet need. Our previous study has identified DKK3 as a potential tumor suppressor in gallbladder cancer, which affects cell invasion, proliferation, and colony forming abilities in GBC cell lines [20]. To further gain deeper insights into the DKK3 signaling network, we overexpressed DKK3 in 3 GBC cell lines and carried out quantitative proteomics and phospho-proteomics analysis using TMT labeling and IMAC (immobilized metal affinity chromatography) based phosphopeptide enrichment. Quantitative proteomic analysis of TGBC24TKB, OCUG-1, G-415 control and DKK3+ cells identified 4646, 4717, and 4419 proteins, respectively. Quantitative phosphoproteomic analysis led to identification of 2382, 3615, and 5518 unique peptides with PTM modifications corresponding to 1110, 1513, and 2006 proteins in TGBC24TKB, OCUG-1 and G-415 cell lines, respectively. To our knowledge, this is the first integrative proteomics and phosphoprotemics profiling of GBC cells lines upon DKK3 overexpression, which would provide a global picture to identify the kinase-driven signaling events when we restore a tumor suppressor gene. In addition, our study led to the identification and predicted activation of several novel molecules that have not been previously associated with DKK3.

## 2. Materials and Methods

### 2.1. Cell Lines and Culture Conditions

3 gallbladder cancer cell lines TGBC24TKB, OCUG-1, G-415 were grown and maintained in DMEM (dulbecco’s modified eagle’s medium) supplemented with 10% fetal bovine serum (FBS) at 37 °C in a humidified atmosphere containing 5% CO_2_.

#### Transient Transfection of *pCS2-hDKK3*

TGBC24TKB, OCUG-1, G-415 were used to determine the optimum concentration of *pCS2-hDKK3* (Addgene catalogue No. 15496) and X-tremeGENE HP DNA transfection reagent (Roche catalogue No. 06366244001). Briefly, DNA was diluted in opti-MEM to a final concentration of 1 µg plasmid DNA per 100 µL medium (0.01 µg/µL) and mixed gently. X-tremeGENE HP DNA transfection reagent was added in 1:2 (DNA:reagent) ratio directly into the medium containing the diluted DNA without coming into contact with the walls of the tube. The complex was mixed gently and incubated for 15 min and added to the cell lines with swirling the wells to ensure even distribution over the entire plate surface [20]. Cell lines were grown in DMEM with 10% FBS for 72 h. Post 72 h, cells were serum starved for 12 h.

### 2.2. Western Blot

Part of transiently overexpressed cell lines: TGBC24TKB, OCUG-1, G-415 were harvested using RIPA lysis buffer (10 mM Tris pH 7.4, 150 mM NaCl, 5mM EDTA (ethylenediaminetetraacetic acid), 1% Triton-X-100, 0.1% SDS containing protease and phosphatase inhibitor cocktails) after serum starvation and sonicated to extract proteins. Western blot analysis was performed, as previously described, using 30 µg protein lysates [21]. Nitrocellulose membranes were hybridized with primary antibodies and developed using Luminol reagent (Santa Cruz Biotechnology, Dallas, TX, USA) as per the manufacturer’s instructions. Anti- beta-actin antibody was obtained from Sigma (St. Louis, MO, USA). Anti-DKK3 antibody (catalogue No. 126080) was obtained from Abcam (Cambridge, MA, USA).

### 2.3. Sample Preparation for MS

#### 2.3.1. Cell Lysis, Protein Extraction

Cell lysis and protein extraction was performed as previously described [22]. *pCS2-hDKK3* (Addgene catalogue No. 15496) was overexpressed transiently in TGBC24TKB, OCUG-1, G-415 cells for 72 h. The cells were serum starved in serum-free medium for 12 h, and then lysed in cell lysis buffer (2% SDS in 50 mM TEABC (triethyl ammonium bicarbonate). Protein concentration was estimated using the BCA method (Pierce; Waltham, MA, USA). Equal amount of protein from control and overexpressed TGBC24TKB, OCUG-1, G415 cells was precipitated using ice-cold acetone.

#### 2.3.2. In-Solution Digestion and TMT Labelling

Proteins were reduced and alkylated using 5mM DTT (dithiothreitol) and 20 mM IAA (iodoacetamide), respectively. Samples were then digested overnight at 37 °C using trypsin (1:20) [23]. Digested peptides were purified using Sep-Pak C18 material. Peptides from the TGBC24TKB, OCUG-1, G-415 cells were labeled using TMT as per the manufacturer’s instruction (Thermo Fisher Scientific, catalogue No. 90110). Peptides from each control condition were labeled with TMT tags as 126, 127N, 127C, and DKK3 overexpressed conditions with TMT tag as 128N, 130N, 131. The labelled peptides were mixed together and 10% were used for the proteome analysis. The remaining 90% sample were used for the IMAC based phosphopeptide enrichment.

#### 2.3.3. Hp-RP StageTip Fractionation Using Copolymer-Based SDB-XC Separation for the Proteome Sample

The TMT labeled peptides were subjected to stage tip based SDB-RP (styrenedivinylbenzene-reverse phase sulphonate) fractionation to generate 3 fractions, as described previously [24]. For packing stage tip 3M™ Empore™ SDB-XC extraction disks were used. TMT labeled peptides were loaded on the disk at 1% TFA (trifluoroacetic acid) and washed with 0.2% TFA. After washing, the peptides were fractionated by the different gradient of ammonium formate as described earlier [24]. For proteomic analysis, 3 fractions were collected and vacuum dried.

#### 2.3.4. IMAC Beads Based Phosphopeptides Enrichment

IMAC beads were prepared from Ni-NTA superflow agarose beads (Qiagen, catalogue No. 1018611) that were stripped of Nickel with 100 mM EDTA and incubated in an aqueous solution of 10 mM FeCl_3_ (Sigma catalogue No. 451649). Dried peptide fractions were reconstituted in 80% acetonitrile/0.1% TFA. Peptide mixtures were enriched for phosphorylated peptides with 10 µL IMAC beads for 40 min. Enriched IMAC beads were loaded on Empore C18 silica packed stage tips. Stage tips were equilibrated with methanol followed by 50% acetonitrile in 0.1% formic acid and 1% formic acid. The beads with enriched peptide were loaded onto C18 stage tips and washed with 80% acetonitrile in 0.1% trifluoroacetic acid. Phosphorylated peptides were eluted from IMAC beads with 500 mM dibasic sodium phosphate, pH 7.0 (Sigma catalogue No. S9763).

### 2.4. LC-MS/MS Analysis

Enriched phosphopeptides and peptides were analyzed on an Orbitrap Fusion Tribrid mass spectrometer (Thermo Electron, Bremen, Germany) interfaced with Easy-nLC II nanoflow liquid chromatography system (Thermo Scientific, Odense, Denmark). Peptides were separated on an analytical column (75 µm × 50 cm, RSLC (rapid separation liquid chromatography) C18) at a flow rate of 280 NL/min using a step gradient of 8–22% solvent B (0.1% formic acid in 90% acetonitrile) for first 70 min, followed by 22–35% up to 103 min. The total run time was set to 120 min. The mass spectrometer was operated in a data-dependent acquisition mode. A survey full scan MS (from m/z 350–1600) was acquired in the Orbitrap at a resolution of 120,000 at 200 m/z. The AGC (automatic gain control) target for MS1 was set as 4 × 10^5^ and ion filling time set 50 milliseconds. The most intense ions with charge state ≥2 was isolated and fragmented using HCD (higher collision dissociation) fragmentation with 34% normalized collision energy and detected at a mass resolution of 50,000 at 200 m/z. The AGC target for MS/MS was set as 1 × 10^5^ and ion filling time set 100 milliseconds dynamic exclusion was set for 30 s.

### 2.5. Data Analysis

The mass spectrometry raw data were searched using Sequest HT search engines with Proteome Discoverer 2.1 (Thermo Fisher Scientific). Phosphopeptide-enriched fractions from each replicate were searched against the RefSeq protein database (version 89). The search parameter used as carbamidomethylation of cysteine residues as a fixed modification. Oxidation of methionine, the phosphorylations of serine, threonine, and tyrosine, were selected as dynamic modifications. Trypsin was set as the protease and a maximum of two missed cleavage were allowed. Precursor mass tolerance was set to 10 ppm, and a fragment mass tolerance of 0.05 Da was allowed. All peptide-spectrum matches (PSM) were identified at a 1% false-discovery rate (FDR). The probability of phosphorylation for each site was calculated by the phosphoRS node in Proteome Discoverer. Only phosphopeptides with >75% site localization probability were considered for further analysis. A 1.5-fold cut-off was used for dysregulated phosphopeptides compared to control cell line. High-confidence PTMs were profiled using PTM-Pro tool [25].

### 2.6. Ingenuity Pathway Analysis (IPA)

The core and comparison analysis were generated using IPA (QIAGEN Ingenuity Pathway Analysis (QIAGEN IPA). Available online: https://www.qiagenbioinformatics.com/products/ingenuity-pathway-analysis (accessed on 15 August 2020)). Significantly altered phosphopeptides in all the 3 cell lines were used to do the analysis. To generate the core analysis, Ingenuity Knowledge Base (genes only) repository was used. Core analysis was created using direct and indirect relationships and species human criteria. The cutoffs were set to 0.58 and −0.58 for hyperphosphorylated and hypophosphorylated molecules respectively. The rest of the filters were set to default to avoid loss of information and relationships. Core analyses identified the top canonical pathways, upstream regulators, etc., significantly enriched in our datasets. After performing core analysis for each cell line, comparison analysis was performed on the output of core analysis for all the 3 cell lines. The analysis results were schematically replicated using Adobe Illustrator (San Jose, CA, USA).

### 2.7. Kinome Map

The KinMap tool (KinMap beta. Available online: http://www.kinhub.org/kinmap/index.html (accessed on 9 June 2020)) was used to build the kinome map. The list of identified kinases were plotted and highlighted on the kinome map as described previously [26]. Dysregulated kinases (hyper phosphorylated or hypo phosphorylated in any cell line) are also depicted.

### 2.8. Kinase-Substrate Enrichment Analysis

The kinase-substrate enrichment analysis was done using the online KSEA tool (KSEA App Kinase-Substrate Enrichment Analysis. Available online: https://casecpb.shinyapps.io/ksea/ (accessed on 1 August 2020)) [27]. Proteins with differentially phosphorylated sites in all the 3 cell lines were used for the input. Kinase-Substrate (K-S) searches were restricted to the PhosphoSitePlus (PSP) database and NetworKIN predictions. Kinases with 1+ substrates are reported, and their respective *z*-scores are reported. NetworKIN predictions with scores 2 or greater. A negative score corresponds to a collective dephosphorylation of the kinase’s substrates; the inverse is true for a positive score. Bars that are colored blue or red are kinases whose score met the *p* < 0.05 score cutoff.

## 3. Results

### 3.1. Proteomic Analysis of DKK3 Overexpressed Cell Lines

**Figure 1:**
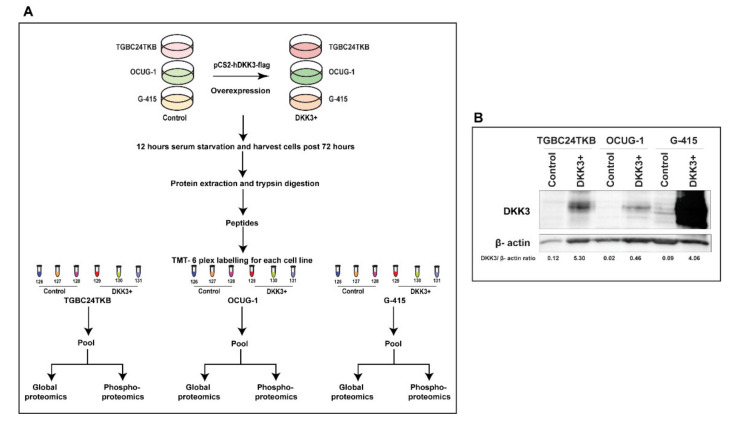
(**A**) Workflow depicting the sample preparation for the study (**B**) Western blot depicting transient transfection of DKK3 in TGBC24TKB, OCUG-1 and G-415 shows the overexpression.

Our previous study [20], identified DKK3 as potential tumor suppressor in gallbladder cancer. However, the downstream regulators of DKK3 are not well studied. To identify downstream regulators of DKK3 signaling pathway, we transiently overexpressed DKK3 in 3 gallbladder cancer cell lines TGBC24TKB, OCUG-1, and G-415 cell lines. These 3 cell lines were chosen based on their invasive property; TGBC24TKB is characterized as non-invasive, OCUG1 is characterized as moderately invasive and G415 as highly invasive GBC cell line. We identified the altered proteome of DKK3 overexpressed cells compared to control cells using TMT-based quantitative proteomics. The experimental workflow is depicted in Figure 1A. Western blot analysis showed relative increase in DKK3 expression in TGBC24TKB, OCUG-1, and G-415 cells compared to control cells (Figure 1B).

**Figure 2:**
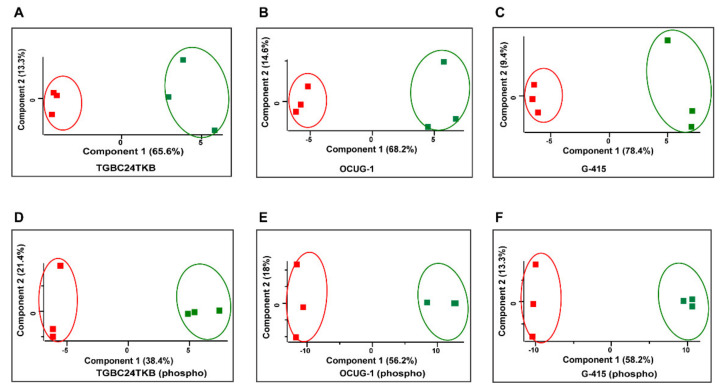
Principal component analysis plot depicting the variation between control and DKK3 overexpressed cells across triplicates in total proteome (**A**–**C**) and in phospho-proteome (**D**–**F**) for TGBC24TKB, OCUG-1, and G-415 cell lines.

Quantitative proteomic analysis of TGBC24TKB, OCUG-1, G-415 control, and DKK3+ cells resulted in identification of 4646, 4717, and 4419 proteins, respectively. Overall, 1904, 1800, and 2361 molecules were found to be *p*-value significant in total dataset. Out of these proteins, 12, 41, 47 of molecules were found to be significantly up-regulated upon DKK3 overexpression in TGBC24TKB, OCUG-1, G-415 cells, respectively, compared to control cells. In addition, 6, 7, 27 of molecules were found to be significantly down-regulated upon DKK3 overexpression in TGBC24TKB, OCUG-1, G-415 cells, respectively, compared to control cells. The list of dysregulated proteins for each cell line are shown in Supplementary Table S1A–C. Principal component analysis also exhibited control and DKK3 overexpressed proteins clustered distinctly as represented in Figure 2. The perceptible clustering may be a consequence of cellular responses elicited by DKK3 overexpression at total (Figure 2A–C) and phosphoproteome (Figure 2D–F) levels in GBC cells.

### 3.2. Phosphoproteomic Analysis of DKK3 Overexpressed Cells

**Figure 3:**
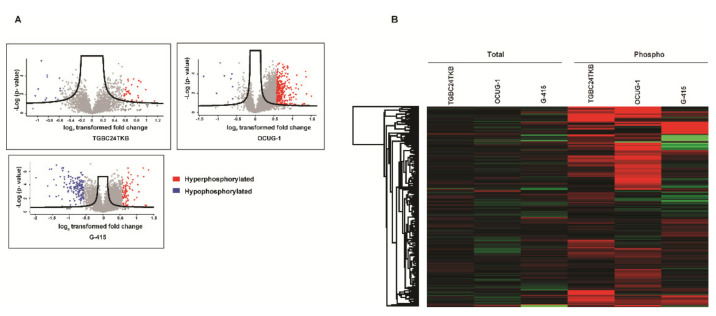
(**A**) Volcano plots showing distribution of molecules in phosphor- proteome for TGBC24TKB, OCUG-1, and G-415 cell lines (**B**) Heatmap depicting the foldchange value across total and phosphoproteins quantified in all 3 cell lines.

The TMT labeled peptides from control and DKK3 overexpressed cells were enriched using IMAC beads as per manufacturers’ protocol and LC MS/MS analysis was carried out as described in methods section. To increase the reliability of our phosphoproteomic analyses, we included technical replicates in our study. The MS data were processed and searched against databases by SEQUEST-HT algorithms using the Proteome Discoverer 2.1 platform. The probability of Ser/Thr/Tyr phosphorylation sites on each peptide was calculated by the PTM-pro algorithm using a cut-off of >75%. We identified 2382 unique peptides with PTM modifications corresponding to 1110 proteins in TGBC24TKB, with a total of 2130 phosphosites identified: 1907 Ser residues, 211 Thr residues, and 12 Tyr residues (Supplementary Figure S1A). Overall, 3615 unique peptides with PTM modifications, corresponding to 1513 proteins in OCUG-1, with a total of 3348 phosphosites identified: 2967 Ser residues, 363 Thr residues, and 18 Tyr residues (Supplementary Figure S1B). 5518 unique peptides with PTM modifications corresponding to 2006 proteins in G-415, with a total of 4744 phosphosites identified: 4227 Ser residues, 501 Thr residues, and 16 Tyr residues (Supplementary Figure S1C). Out of these molecules, 30 (40 phosphosites corresponding to 30 proteins), 202 (289 phosphosites corresponding to 202 proteins), 60 (76 phosphosites corresponding to 60 proteins) were significantly hyperphosphorylated in TGBC24TKB, OCUG-1, and G-415 cell lines, respectively. Additionally, 12 (13 phosphosites corresponding to 12 proteins), 8 (8 phosphosites corresponding to 8 proteins), 138 (141 phosphosites corresponding to 138 proteins) were significantly hypophosphorylated in TGBC24TKB, OCUG-1, and G-415 cell lines, respectively. The list of hyperphosphorylated and hypophosphorylated molecules for each cell line are shown in Supplementary Table S2A–C. Volcano plots representing fold change distribution of phospho proteins identified in TGBC24TKB, OCUG-1, G-415 cell lines are depicted in (Figure 3A). The heatmap between the quantified molecules across all 3 cell lines at total and phosphoproteome represents that majority of molecules were dysregulated at phosphorylation level and not at basal or total levels only (Figure 3B).

### 3.3. Phosphoproteomic Analysis Identified Alteration in Signaling Pathways in DKK3 Overexpressed Cells

**Figure 4:**
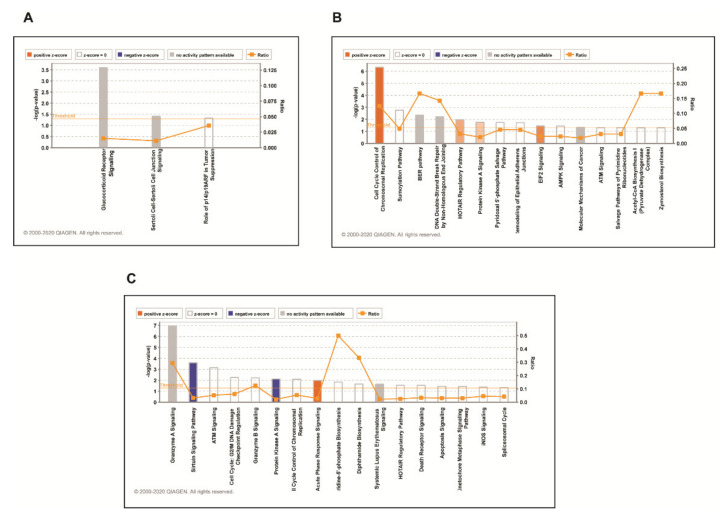
Top canonical pathways enriched in DKK3 overexpressed cells in phospho-proteome for (**A**) TGBC24TKB (**B**) OCUG-1 and (**C**) G-415 cell line.

To understand the biological insights of DKK3 signaling, we utilized an ingenuity pathway analysis tool to analyze phosphoproteomic data. We considered proteins that exhibited differential phosphorylation by 1.5-fold in each cell line to identify enriched biological processes. We observed that DKK3 overexpression led to differential phosphorylation of proteins involved in different biological processes in all 3 cell lines. The top canonical pathways identified in TGBC24TKB cells were Glucocorticoid Receptor Signaling (*p*-value 2.5 × 10^−4^), Sertoli Cell-Sertoli Cell Junction Signaling (*p*-value 3.88 × 10^−2^), Role of p14/p19ARF in Tumor Suppression (*p*-value 4.72 × 10^−2^) (Figure 4A). The top canonical pathways identified in OCUG-1 cell line were Cell Cycle Control of Chromosomal Replication (*p*-value 4.71×10^−7^), Sumoylation Pathway (*p*-value 1.74 × 10^−3^), BER pathway (*p*-value 4.50 × 10^−3^), DNA Double-Strand Break Repair by Non-Homologous End Joining (*p*-value 6.14 × 10^−3^), HOTAIR Regulatory Pathway (*p*-value 1.09 × 10^−2^) (Figure 4B). The top canonical pathways identified in G-415 cell line were Granzyme A Signaling (*p*-value 1.15 × 10^−7^), Sirtuin Signaling Pathway (*p*-value 2.49 × 10^−4^), ATM Signaling (*p*-value 7.11 × 10^−4^), Cell Cycle: G2/M DNA Damage Checkpoint Regulation (*p*-value 5.57×10^−3^), Granzyme B Signaling (*p*-value 6.00 × 10^−3^) (Figure 4C).

### 3.4. DKK3 Overexpression has Differential Effect on Cell Lines with Respect to Invasiveness as Predicted by Ingenuity Pathway Analysis

**Figure 5:**
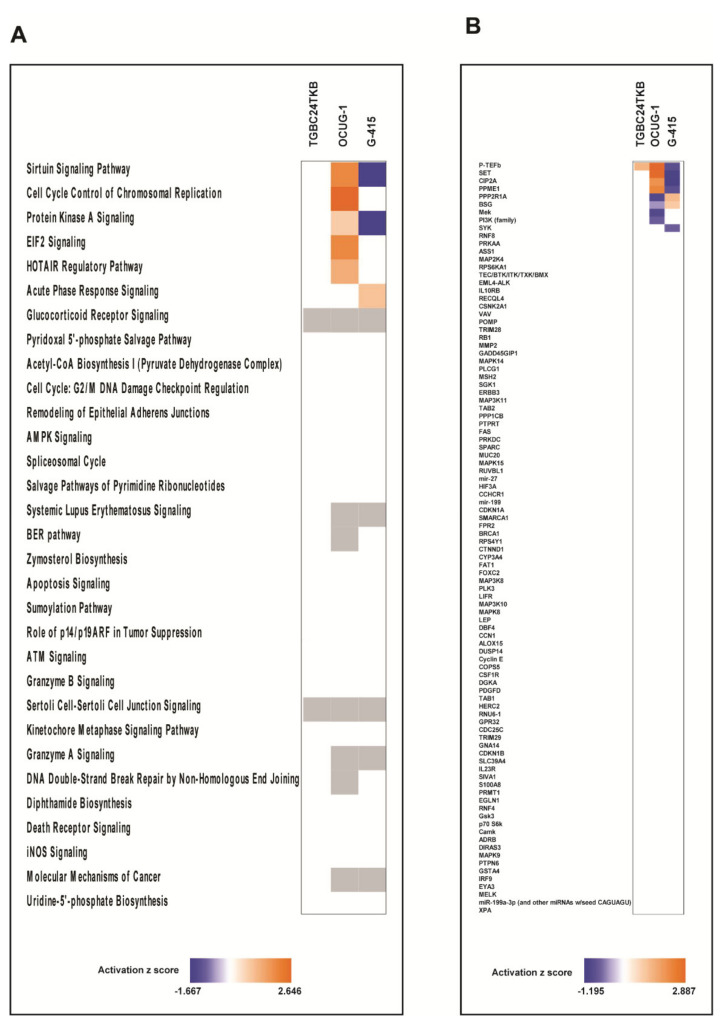
(**A**) Heatmap depicting the differentially activated pathways across 3 cell lines (**B**) Heatmap depicting the differentially activated regulators across 3 cell lines.

**Figure 6:**
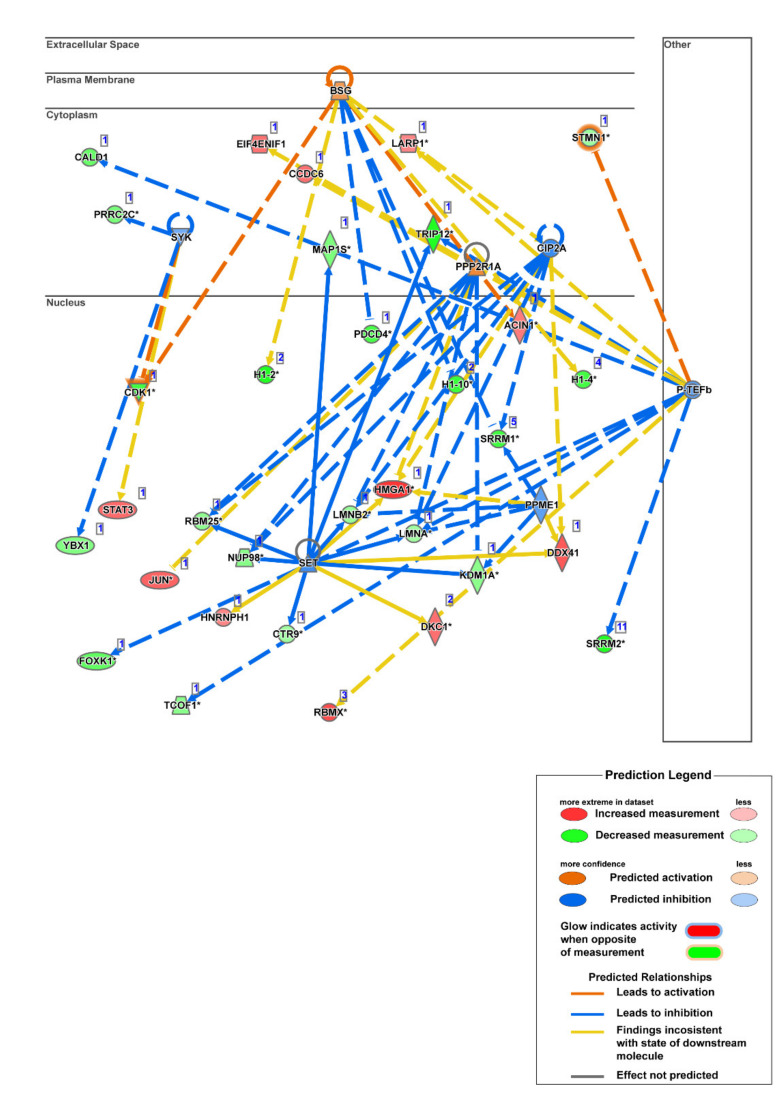
Schematic diagram for networks sets of connected upstream regulators that can work together to elicit the expression changes observed in G-415 dataset.

To further compare the observations of DKK3 overexpression on cell lines with varying invasiveness properties, we applied the comparison analysis from IPA. To our interest we observed most of the pathways were unchanged in TGBC24TKB, the least invasive cell line. However, DKK3 overexpression showed differential effect on moderately invasive and highly invasive cell lines. Overexpression of DKK3 activated Sirtuin Signaling Pathway in OCUG1 (*z* score = 2) and inactivated in G-415 (*z* score = −1.667). Simultaneously, pathway leading to Cell Cycle Control of Chromosomal Replication was activated only in OCUG1 cell line with *z* score of 2.646. The Protein Kinase A Signaling pathway was activated in OCUG-1 (*z* score = 0.816) and inactivated in G-415 (*z* score = −1.633) (Figure 5A). To further deepen our understanding that can explain the differential effect of DKK3, we applied the Upstream regulatory analysis (URA) from IPA which identifies the upstream molecules that can affect the expression of other molecules. Our analysis predicted set of upstream regulators which showed differential activation with respect to invasiveness. P-TEFb (positive transcription elongation factor-beta), was predicted to be activated in TGBC24 and OCUG1 and inhibited in G-415 cells. Similarly, SET (phosphatase 2A inhibitor I2PP2A), CIP2A (cancerous inhibitor of protein phosphatase 2A), PPME1 (protein phosphatase methylesterase 1) were predicted to be activated in OCUG1 cells and inhibited in G-415 cells. However, the molecules PPP2R1A (protein phosphatase 2 scaffold subunit alpha) and BSG (basigin) were predicted to be inhibited in OCUG1 cells and activated in G-415 cells (Figure 5B). The mechanistic networks set of connected upstream regulators that can work together to elicit the expression changes observed in highly invasive G-415 cells is depicted in Figure 6 and for OCUG-1 and TGBC24TKB in Supplementary Figures S2 and S3.

### 3.5. Kinases Enriched in Phosphoproteomic Dataset upon DKK3 Overexpression

**Figure 7:**
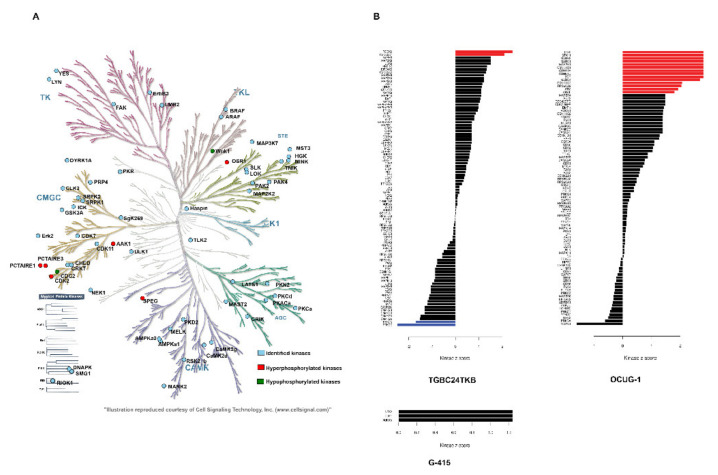
(**A**) Kinome map depicting the identified kinases in the dataset. Kinases are highlighted as blue: identified kinases; red: hyperphosphorylated kinases; green: hypophosphorylated kinases. The map was built using the KinMap tool (**B**) Predicted upstream kinases enriched in GBC cell lines. Bars that are colored blue/red are kinases whose score met the *p* < 0.05 score cutoff.

In our phosphoproteomic dataset, we identified 79 non redundant kinases. In TGBC24TKB, 13 kinases were identified. In OCUG-1, 45 kinases were identified of which, 10 were hyperphosphorylated. In G-415 cell line, 49 kinases were identified, out of which 1 was hyperphosphorylated and 5 were hypophosphorylated. The list of dysregulated kinases along with their phosphorylation sites are provided in Table 1. We mapped all the identified kinases in KinMap tool for better visualization of the identified kinases. Our analysis led to mapping of 61 out of 79 kinases in KinMap tool for all 3 cell lines. Out of 61 kinases, 16 belonged to CMGC (which refers to the CDK, MAPK, GSK3, and CLK set of families) family; 11 belonged to the homologs of yeast Sterile 7, Sterile 11, and Sterile 20 kinases (STE) family; 7 belonged to protein kinase A, G, and C family (AGC); 9 belonged to calmodulin/calcium regulated kinases (CAMK) family; 5 belonged to tyrosine kinases (TK) family; 2 belonged to tyrosine-like kinases (TKL) family; none belonged to casein kinase family (CK1); 8 were from other families and 3 were atypical kinases. Overall, DKK3 overexpression led to hyperphosphorylation of 11 kinases and hypophosphorylation of 5 kinases in at least one cell line (Figure 7A). Further to understand the kinase activity upon DKK3 overexpression we performed KSEA analysis. Our analysis identified that substrates for ROCK2 (Rho associated coiled-coil containing protein kinase 2), CSNK2A1(casein kinase 2 alpha 1) kinases were hyperphosphorylated and that substrates for PRKCA, PRKCE kinases were hypophosphorylated significantly in TGBC24TKB. Similarly, the substrates for TLK2(tousled like kinase 2), STK11 (serine/threonine kinase 11), MARK3 (microtubule affinity regulating kinase 3), MARK1 (microtubule affinity regulating kinase 1), MAP2K1 (mitogen-activated protein kinase kinase 1), CSNK1G1 (casein kinase 1 gamma 1), CSNK1E (casein kinase 1 epsilon), CSNK1D (casein kinase 1 delta), BCR (BCR activator of RhoGEF and GTPase), MARK4 (microtubule affinity regulating kinase 4), CSNK1G3 (casein kinase 1 gamma 3), RPS6KA2 (ribosomal protein S6 kinase A2), ATM (ATM serine/threonine kinase), ARAF (A-Raf proto-oncogene, serine/threonine kinase) kinases were hyperphosphorylated significantly in OCUG1 cell line. However, we failed to observe any kinases that showed significant substrate phosphorylation or dephosphorylation in G-415 cell line (Figure 7B). The kinase-substrate links are provided in Supplementary Table S3.

## 4. Discussion

DKK3 also known as REIC plays an important role in several biological processes. Dysregulated DKK3 expression is linked to the development of a number of diseases like cardiac hypertrophy [28], systemic lupus erythematosus [29], Alzheimers [30], bone formation and bone diseases [31], cancer [7,8,9], etc. Current understanding of the molecular mechanism of DKK3 signaling is limited. This study provides the first unbiased and quantitative investigation of the proteome and phosphoproteome and its dynamic changes in response to DKK3 overexpression in GBC cell lines. We employed a TMT-based labeling approach coupled with IMAC-based phosphopeptide enrichment coupled to high-accuracy mass spectrometry to reproducibly identify and quantify a large number of serine, threonine, and tyrosine phosphorylation sites with high confidence.

Multiple groups have applied conventional approaches to study the effects of DKK3 in different model organisms, and there are no studies till date which have analyzed downstream effects of DKK3 using high throughput platform. Our proteomics and phosphoproteomics data analysis revealed that alteration was majorly seen at phosphorylation level upon DKK3 overexpression in gallbladder cancer cells. To understand the downstream dysregulated pathways, we analyzed phosphoproteomic data for all the 3 cell lines distinctively. Ingenuity pathway analysis identified molecules based on their functional annotation were significantly associated with 9 molecular and cellular functional categories; RNA Post-Transcriptional Modification, Cellular Development, Cell Death and Survival, Protein Synthesis, Cell Morphology, Cellular Assembly and Organization, Cell Cycle, Gene expression, and Cellular Growth and Proliferation (Table 2). DKK3 is widely linked to cellular development in kidney [32], heart [5], prostate [33], etc. Multiple groups have also associated DKK3 with cell death and survival. Abarzua et al. observed that ectopic Dkk-3 expression leads to apoptosis via c-Jun NH2-terminal kinase (JNK) in prostate cancer cells [34]. In Non-small cell lung cancer (NSCLC) cells, it was observed that DKK3 overexpression inhibits the growth of NSCLC cells by inducing apoptosis and cell cycle disturbance by transactivating c-myc and cyclin D1 through β-catenin/TCF-4 signaling [35]. Xiang et al. has observed effect of DKK3 transfection on cell morphology which led cells to regain cell–cell contacts and maintain adherence in MB231 and BT549 cell lines [36]. In our previous study, we had identified tumor suppressive role of DKK3 in GBC cell lines [20]. We observed effects of DKK3 overexpression on the proliferative, invasive, and colony forming abilities of GBC cell lines.

The IPA analysis compared the effect of DKK3 overexpression with respect to the invasiveness of the cell lines. Our comparison analysis identified the set of pathways predicted to be differentially activated in GBC cell lines. We identified several pathways which are not linked with the expression of DKK3 before. The Sirtuin signaling pathway and Protein kinase A signaling pathway were predicted to be activated in OCUG-1 cell line compared to G-415 cell line. However, the exact role of DKK3 in these pathways is not fully understood. Among the list of upstream regulators, we identified novel molecules that showed differential activation and are never linked to DKK3 expression before. From our analysis, we identified molecules such as P-TEFb, PPME, SET, CIP2A which showed differential activation pattern upon DKK3 overexpression with respect to the invasiveness of the cell lines. P-TEFb is a kinase that phosphorylates the C-terminal domain of the large subunit (RPB1) of RNA polymerase II and subsequently stimulates productive transcription elongation of multiple cellular genes. It is a master regulator involved in transcription elongation and its dysregulation is involved in multiple diseases including cancer. P-TEFb release and activation by CDK9 inhibitors or by other releasers and activators is known to promote cell growth arrest [37]. Activation of P-TEFb upon DKK3 overexpression further strengthens its role as potential tumor suppressor. PP2A is one of the chosen targets by viruses to manage host cell machinery and program cells into malignant state [38]. With a general view of PP2A as tumor suppressor, it is also clear that, in some signaling steps, PP2A acts as a positive factor for cell growth and survival. CIP2A, PPME, and SET are PP2A inhibitor proteins (PAIP) and have been largely involved in suppression of PP2A function through different mechanisms [39]. Inhibition of these PAIPs in G-415 cell line largely supports the role of DKK3 in regulation of PP2A and its combined role in tumor suppression is further warranted.

We further identified potential altered kinases upon DKK3 overexpression that could be validated to deduce the mechanism by which DKK3 acts. Among all the dysregulated kinases, CDKs (cyclin-dependent kinases) were majority. It is well established, a fundamental aspect of cancer is dysregulated cell cycle control and CDK being a major part regulating cell cycle control, our data identified 6 CDKs to be differentially phosphorylated. Among the differentially phosphorylated CDK’s CDK1 is only the kinase, which was hypophosphorylated in our data while CDK16, CDK11A/B, CDK2, and CDK18 were hyperphosphorylated. The exact mechanism for the differential phosphorylation for these kinases needs to be validated. Nevertheless, the result of these alterations is important in studying the tumor suppression function of DKK3. We also observed hyperphosphorylation of serine/threonine-protein kinase PRP4 homolog (PRPF4B) at 3 different positions S257, S292; S294, which is involved in pre-mRNA splicing. We also observed the hyperphosphorylation of kinase involved in myocyte cytoskeletal development: (SPEG), Oxidative Stress Responsive Kinase 1 (OXSR1), etc. A consolidated list for the available small molecule inhibitors that target against dysregulated kinases was adapted from Moret et al., and is depicted in Supplementary Figure S4 [40].

Overall, the data generated from this study greatly expands the understanding of the downstream modulators upon DKK3 overexpression. Our study highlights the list of dysregulated molecules to be important in regulation of various process initiated by overexpressing DKK3. For the very first time the complexities and responses involved upon DKK3 overexpression and its effect on biological functions and cellular phenotype are identified using high-throughput method. We have characterized differential protein expression and phosphorylation events that occur upon DKK3 overexpression. We identified differential activation of regulators and pathways that hold potential significance with respect to DKK3 signaling and needs to be unfolded in time ahead. Our findings help in unraveling the complex biological nature of DKK3 molecule. However, as the next step of this study, a detailed analysis of the molecules identified in the study is needed, which is indeed a limitation for this study. Our study highlights downstream effect of DKK3 overexpression at global proteomic and phosphoproteomic levels in gallbladder cancer cell lines, however the deeper validation of these integrated signaling molecules would provide a more explicit visualization. Furthermore, assessing the involvement of regulatory networks described in our study needs to be widened across multiple cellular and biological models.

## Figures and Tables

**Table 1:** List of dysregulated kinases identified upon DKK3 overexpression.

Gene Symbol	Protein Description	PTM Window (PTM-Pro2.0)	PTM_Site (Protein)	Fold Change
CDK16	cyclin-dependent kinase 16	EDINKRLsLPADIRL	S193	2.4
PRPF4B	serine/threonine-protein kinase PRP4	EKIGKARsPTDDKVK	S257	1.7
CDK11A	cyclin-dependent kinase 11A	QQRVKRGtSPRPPEG	T739	1.7
CDK2	cyclin-dependent kinase 2	VEKIGEGtYGVVYKA	T14	1.7
AAK1	AP2-associated protein kinase 1	GQKVGSLtPPSSPKT	T620	1.6
CDK2	cyclin-dependent kinase 2	VEKIGEGtYGVVYKA	T14	1.6
CDK11B	cyclin-dependent kinase 11B	KEKRRHRsHSAEGGK	S113	1.6
PRPF4B	serine/threonine-protein kinase PRP4	SRDRGKKsRSPVDLR	S292	1.6
SPEG	striated muscle preferentially expressed protein kinase	PKTSRAVsPAAAQPP	S559	1.5
CDK18	cyclin-dependent kinase 18	GEEPGQLsPGVQFQR	S142	1.5
OXSR1	serine/threonine-protein kinase OSR1	ALSSGSGsQETKIPI	S480	1.6
PRPF4B	serine/threonine-protein kinase PRP4	EKIGKARsPTDDKVK	S257	0.6
WNK1	serine/threonine-protein kinase WNK1	PPPARSGsGGGSAKE	S185	0.5
CDK1	cyclin-dependent kinase 1 isoform 1	EKIGEGTyGVVYKGR	Y15	0.4
PPP1R12A	protein phosphatase 1 regulatory subunit 12A	STGVSFWtQDSDENE	T898	2.1
PTPRA	receptor-type tyrosine-protein phosphatase alpha	SVPLLARsPSTNRKY	S211	1.6

**Table 2:** Table depicting top Molecular and cellular functions upon IPA analysis.

Function	TGBC24TKB *p*-Value Range	OCUG-1 *p*-Value Range	G-415 *p*-Value Range
RNA Post-Transcriptional Modification	4.39 × 10^−2^–1.20 × 10^−4^	2.50 × 10^−3^–3.06 × 10^−19^	1.54 × 10^−3^–1.49 × 10^−17^
Cellular Development	4.72 × 10^−2^–2.88 × 10^−5^	8.52 × 10^−3^–4.85 × 10^−9^	1.46 × 10^−2^–4.38 × 10^−8^
Cell Morphology	3.73 × 10^−2^–6.35 × 10^−6^	Not significant	Not significant
Cellular Assembly and Organization	4.72 × 10^−2^–8.65 × 10^−6^	Not significant	Not significant
Cell Cycle	4.72 × 10^−2^–2.88 × 10^−5^	Not significant	1.46 × 10^−2^–3.00 × 10^−7^
Cell Death and Survival	Not significant	8.52 × 10^−3^–7.36 × 10^−10^	Not significant
Cellular Growth and Proliferation	Not significant	8.52 × 10^−3^–4.85 × 10^−9^	1.46 × 10^−2^–4.38 × 10^−8^
Protein Synthesis	Not significant	5.84 × 10^−3^–1.36 × 10^−7^	Not significant
Gene expression	Not significant	Not significant	1.46 × 10^−2^–5.08 × 10^−7^

## Data Availability

Not applicable.

## Supplementary Materials

The following are available online at https://www.mdpi.com/2073-4409/10/3/511/s1, Figure S1: Pie chart depicting the phosphosites identified in the study. Identification of the unique phosphosites in percentage. Figure S2: Schematic diagram for networks sets of connected upstream regulators that can work together to elicit the expression changes observed in OCUG-1 dataset. Figure S3: Schematic diagram for networks sets of connected upstream regulators that can work together to elicit the expression changes observed in TGBC24TKB dataset. Figure S4: Schematic diagram depicting available small molecule inhibitors for dysregulated kinases. Multiple kinase-targeting small molecules are color coded for better visualization. Table S1A: Dysregulated proteins identified in TGBC24TKB cell line. Table S1B: Dysregulated proteins identified in OCUG-1 cell line. Table S1C: Dysregulated proteins identified in G-415 cell line. Table S2A: Dysregulated phospho proteins identified in TGBC24TKB cell line. Table S2B: Dysregulated phospho proteins identified in OCUG-1 cell line. Table S2C: Dysregulated phospho proteins identified in G-415 cell line. Table S3: List of Kinase-Substrate Links adapted from KSEA tool.
