# Supplementary material for: Integrated Proteomic and Phosphoproteomics Analysis of DKK3 Signaling Reveals Activated Kinase in the Most Aggressive Gallbladder Cancer
Source: Cells. 2021 Feb 28;10(3):511. doi: 10.3390/cells10030511 (PMC7997438; doi:10.3390/cells10030511)

Supplementary Figure S1

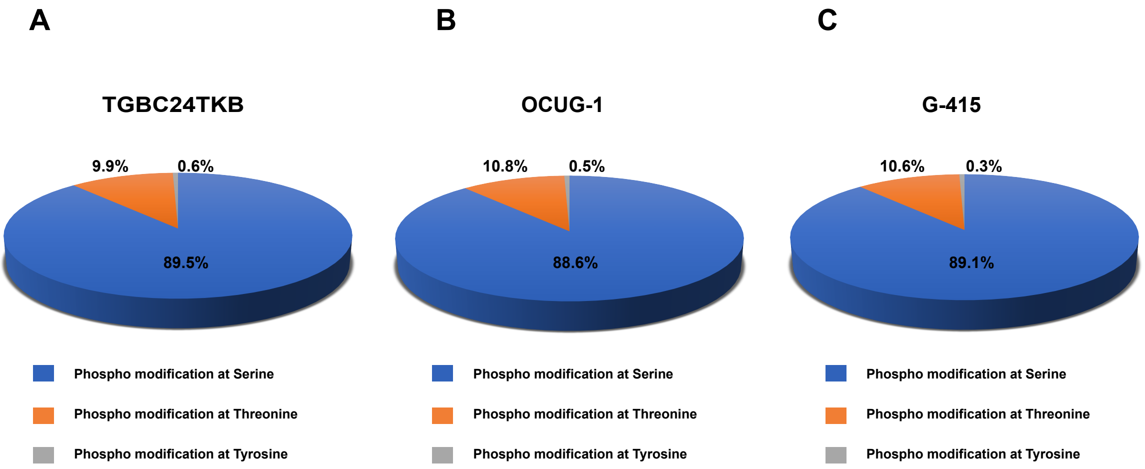

Supplementary Figure S2

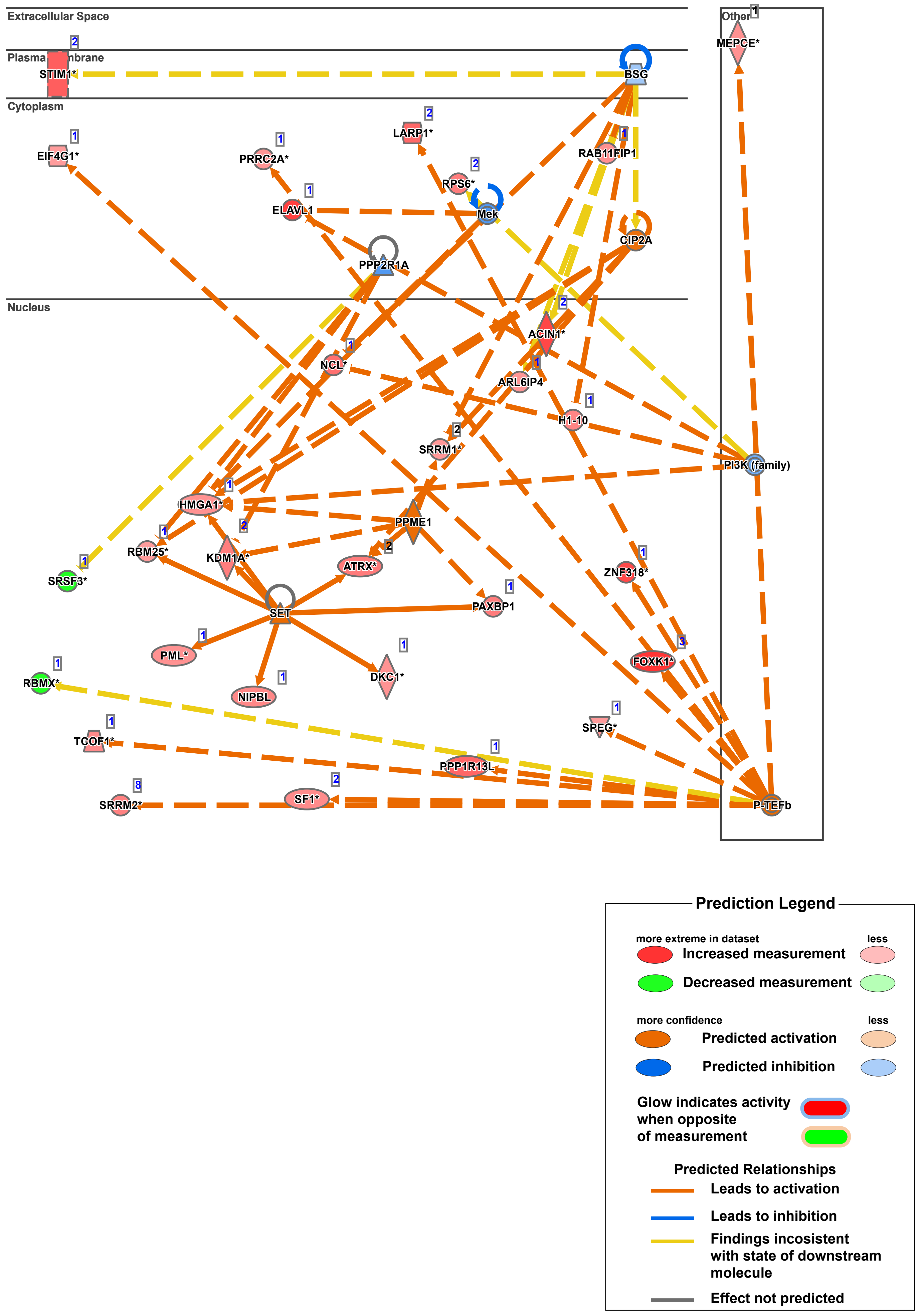

Supplementary Figure S3

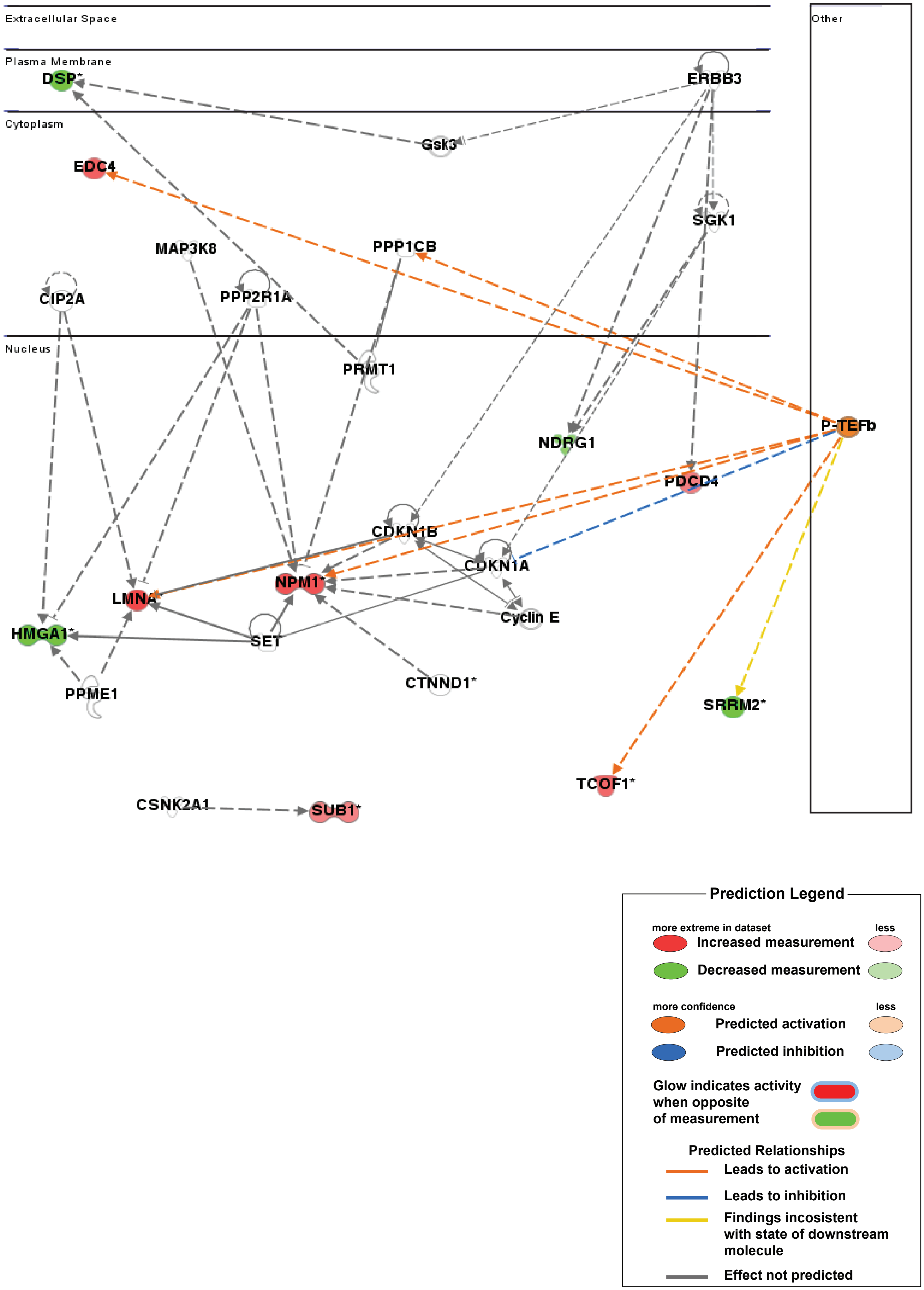

Supplementary Figure S4

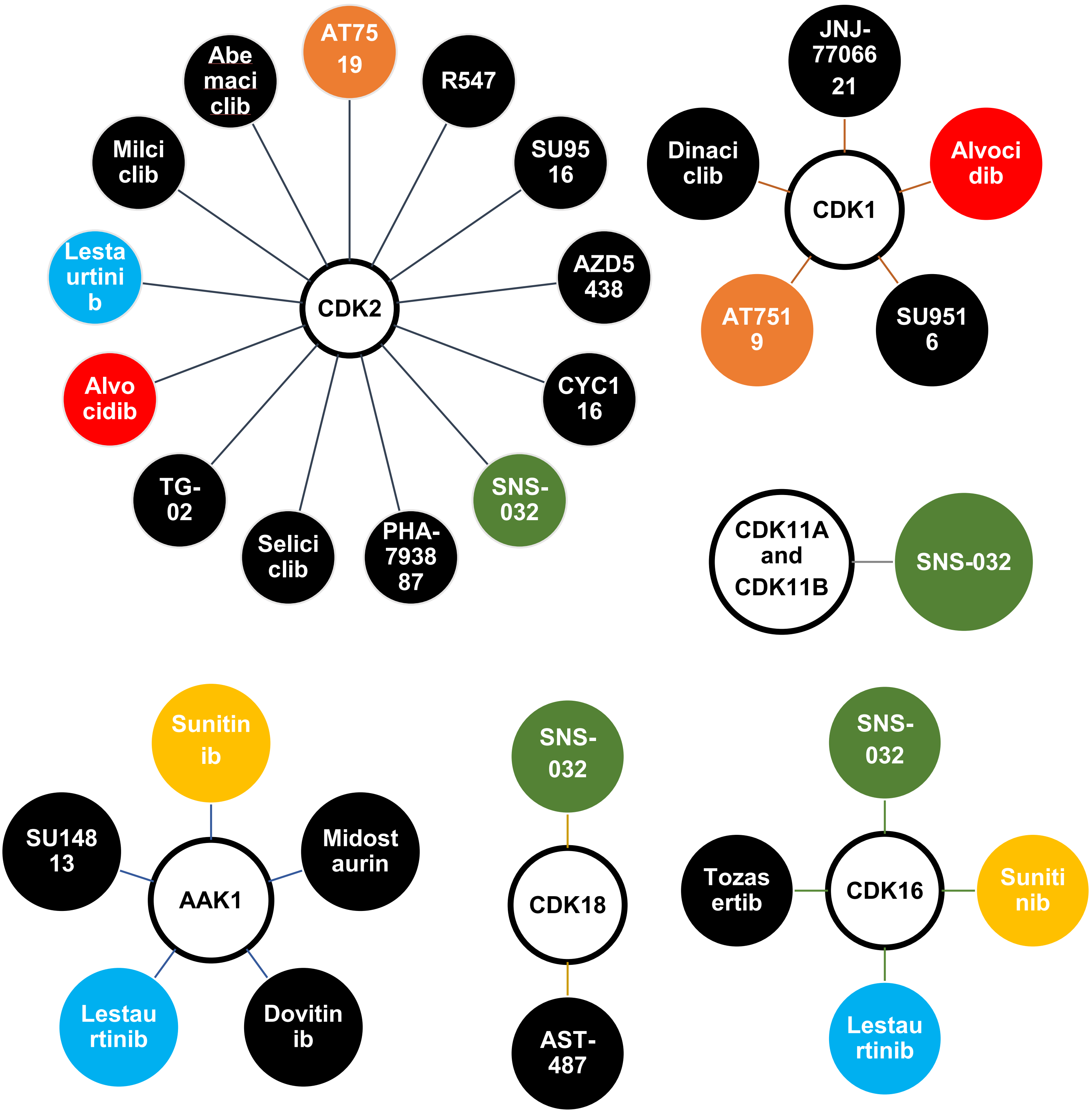

Supplement: Supplementary file 1 [file cells-10-00511-s001.zip › Gondkar et al_Revised Supplementary Figures_012521.pdf]
